# Year in review 2013: *Critical Care* - respirology

**DOI:** 10.1186/s13054-014-0577-y

**Published:** 2014-10-15

**Authors:** Gerard F Curley, Arthur S Slutsky

**Affiliations:** Department of Anesthesia, St Michael’s Hospital, and the Keenan Research Centre for Biomedical Science of St Michael’s Hospital, 30, Bond Street, Toronto, ON M5B 1 W8 Canada; Department of Anesthesia, University of Toronto, 27, King’s College Circle, Toronto, ON M5S Canada; Department of Medicine, St Michael’s Hospital, and the Keenan Research Centre for Biomedical Science, St Michael’s Hospital, 30 Bond Street, Toronto, ON M5B 1 W8 Canada; Interdepartmental Division of Critical Care, University of Toronto, 27, King’s College Circle, Toronto, ON M5S Canada

## Abstract

This review documents important progress made in 2013 in the field of critical care respirology, in particular with regard to acute respiratory failure and acute respiratory distress syndrome. Twenty-five original articles published in the respirology and critical care sections of *Critical Care* are discussed in the following categories: pre-clinical studies, protective lung ventilation – how low can we go, non-invasive ventilation for respiratory failure, diagnosis and prognosis in acute respiratory distress syndrome and respiratory failure, and promising interventions for acute respiratory distress syndrome.

## Introduction

Acute respiratory distress syndrome (ARDS) represents a recognizable common pattern of acute alveolar-capillary injury in critically ill patients. Despite numerous randomized clinical trials aimed at regulating the lung inflammatory response during ARDS [1], the only proven therapies to consistently reduce mortality are a protective ventilation strategy and prone positioning [2,3]. This review outlines the progress made in basic science and clinical respiratory critical care research in 2013, which is likely to further our understanding of pathophysiology in ARDS and acute respiratory failure and potentially identify new therapeutic strategies. This includes pre-clinical investigation, observational studies and meta-analysis, and phase II and III trials.

## Pre-clinical studies

Although we have made great strides in understanding the pathogenesis of respiratory failure in ARDS, we do not yet have sufficient understanding of the underlying mechanisms of alveolar-capillary barrier dysfunction in ARDS [4,5]. To address this, mouse and rat models are commonly used; unfortunately, many are poor models for the majority of human diseases [6]. Crucial genetic, molecular, immunologic, and cellular differences between humans and mice prevent animal models from serving as effective and reliable surrogates of human ARDS [7]. The failure to translate from animals to humans is likely due in part to poor methodology as well as the failure of the models to accurately mimic the human disease condition [8,9]. It has been recommended that experiments be designed in both genders and in different age groups of animals and that all data, both positive and negative, be published [10].

In this respect, a study by Setzer and colleagues [11] evaluated the effects of high-stretch mechanical ventilation on ‘old’ and ‘young’ rats. The conclusion drawn from these experiments was that older rats are more susceptible to injury induced by high tidal volumes, including enhanced leukocyte migration and alveolar-capillary barrier dysfunction. Animal age has been regarded as a confounding factor in pre-clinical experiments in ARDS, given that the evidence for a potential therapeutic is usually derived from experiments in young animals, which are not representative of adult disease. Age is indeed associated with compromised physiologic and immunologic function, even in the absence of disease [12]. Age-dependent changes in respiratory and cardiovascular reserve are well documented [13,14]. The age-dependent deterioration of the immune system results in increased susceptibility to viral and bacterial infections, opportunistic infections, reactivation of latent viruses, decreased responses to vaccination, autoimmune diseases, and neoplasias in humans and animals [15]. In addition, there is an age-dependent systemic inflammatory state in humans and animals even in the absence of disease [16]. The lungs of aged individuals also exhibit an elevated basal inflammatory state [17], which is primed to respond in an overexuberant manner following an infection or injury. Regardless of whether local (organ-specific) or systemic, the elevated inflammatory state is characterized by elevated basal levels of the proinflammatory mediators IL-6, IL-8, IL-1, and tumor necrosis factor-alpha [18].

Thus, Setzer and colleagues confirm that young rats are less susceptible to ventilator-induced lung injury (VILI) than older rats. Failure to adequately consider variables such as age, as well as comorbidity, physiological status, and timing of drug administration, contributes to the disparity between the results of animal models and clinical trials.

Kim and colleagues [19], in an effort to mimic and modify the lung injury that occurs during neutropenia recovery, administered tyrosine kinase inhibitors to mice that had been rendered neutropenic. Indeed, this elegant and physiologically relevant study also identified a separate potential therapeutic target, platelet-derived growth factor (PDGF) receptor β. PDGF has been implicated as a pro-fibrotic stimulus in ARDS [20]. However, the α and β receptors for PDGF mediate different functions: the β receptor is important in mediating pro-fibrotic cell migration, whereas the α receptor inhibits migration. Studies such as these, which identify novel molecular targets in clinically relevant animal models, portend a brighter future for pharmacologic intervention in ARDS.

In another important pre-clinical study, Protti and colleagues [21] evaluated the injurious effects of high positive end-expiratory pressure (PEEP) in healthy lungs during low tidal volume mechanical ventilation. PEEP has become a central component of protective lung ventilation strategies. Although the degree of alveolar over-distention to which animals are subject is central to the pathogenesis of VILI, it also appears likely that unstable lung units in ARDS may be damaged by repeated opening and closing during tidal ventilation [22]. PEEP may prevent diffuse alveolar damage in experimental models during prolonged ventilation at high lung volumes by stabilizing distal lung units [23]. The flip-side of overinflation and overdistension, the potential for low-volume injury, has been addressed by experiments in isolated rat lungs [24] that demonstrated that repetitive opening and collapse can lead to a decrease in lung compliance and injury to the epithelial cells that line small airways and alveolar ducts. Protti and colleagues have addressed the potential of high PEEP to increase alveolar capillary permeability and exacerbate pulmonary edema. Previous studies have shown that only major increases in static lung volume alter epithelial permeability to large molecules during static inflation [25,26]. In contrast, prolonged cyclic lung inflation during mechanical ventilation produces major alterations in epithelial permeability to proteins, both large and small [27]. The study by Protti and colleagues confirms that high PEEP does not increase pulmonary edema in healthy lungs, even after subsequent removal of the PEEP [21].

Finally, Vecchi and colleagues [28] used an ovine oleic acid injury model to determine the effects of lowering radiation dose on the quality of computed tomography (CT) images. This study demonstrated that a reduction of effective radiation dose of up to 70% can be achieved with minimal effects on lung quantitative results and that low-dose CT could therefore be a valuable tool for the characterization of lung compartment distribution and possibly for monitoring the progression of ARDS, with a lower risk of exposure to ionizing radiation. This study has formed the basis for an observational study that reached similar conclusions in patients with ARDS [29].

## Protective lung ventilation – how low should we go

The possibility that mechanical ventilation can actually worsen lung injury is now accepted as reality [30]. More recent attempts to adjust ventilation strategies to further reduce harm have met with limited success [31-33]. Even with contemporary low-stretch strategies, it appears difficult to avoid regional areas of high lung stretch [34] in some patients. Quantitative assessment of CT images in humans with severe ARDS indicates that the amount of normally aerated tissue - the so-called ‘baby lung’ - is variable and may be as low as 200 mL [35]. A 6 mL/kg tidal volume applied to these ‘baby lungs’ results in airway pressures in the range of 30 to 35 cm H_2_O (which in many patients is likely to be injurious) [36]. In this regard, the mean peak airway pressure in the treatment arm of the ARDS net low tidal volume study was 34 cm H_2_O [36]. Other diseased lung regions may be subject to even greater distention and greater regional intra-alveolar and airway pressures [37]. However, further lowering the tidal volume to prevent lung injury may worsen atelectasis [38], which can also cause harm [39].

In this respect, a 2013 pilot randomized crossover study [40], which compared a 4 mL/kg tidal volume mechanical ventilation strategy with 6 mL/kg in patients with ARDS, is promising. Patients who were ventilated with a tidal volume of 4 mL/kg had decreased cyclic recruitment-derecruitment and end-inspiratory hyperinflation on dynamic CT, as well as lower plateau airway pressures, compared with those ventilated with 6 mL/kg. This study is intriguing not only for the potential enhanced lung protection evident on radiologic imaging but also for the questions it poses for the management of the resultant hypercapnia. Hypercapnia is common in ARDS. Managing elevated arterial partial pressure of carbon dioxide (PaCO_2_) by increasing tidal volume is now known to be unacceptable in many situations; however, management by increasing the respiratory rate, as in this study, is common but of uncertain impact. For example, increasing respiratory frequency from 12 to 30 breaths per minute adds over 25,000 additional opening and closing cycles per day to an already injured lung, and laboratory data suggest that this approach can be associated with additional lung injury [41].

The use of extra-corporeal lung support may obviate the need for increased respiratory rate or increased tidal volume, but this therapy remains unproven. Adding to the uncertainty around lowering tidal volumes further is the fact that hypercapnia might contribute direct benefit in patients with ARDS [42]. A multivariate analysis of the ARDS Network low tidal volume study [2], after controlling for other variables predictive of mortality, found that the patients who had moderate hypercapnic acidosis (HCA) (pH 7.15 to 7.35, PaCO_2_ 45 to 65 mm Hg) on study day 1 had a significantly lower odds ratio of death at 28 days, but only in the 12 mL/kg tidal volume group, a result consistent with a protective effect of HCA in VILI [43]. Though not proof of cause and effect, these data support the concept that hypercapnia during low tidal volume ventilation might contribute direct benefit in patients with ARDS.

Another randomized crossover study in 2013 addressed this very point. Natalini and colleagues [44] ventilated 16 patients with ARDS for 30 minutes with either low (6 mL/kg) or high (12 mL/kg) tidal volume ventilation and then obtained hemodynamic measurements, including cardiac index and oxygen delivery. Cardiac index and oxygen delivery index were increased with low compared with high tidal volume ventilation, whereas oxygen extraction ratio decreased. The enhanced cardiac index was positively associated with PaCO_2_ variation and not with alterations in tidal volume or airway pressure.

The potential for harm with hypercapnic or metabolic acidosis is clear, whether due to acute exposure (for example, raised intracranial pressure, pulmonary hypertension) or exposure for prolonged periods of time (for example, increased risk of infection) or at high concentrations. Although HCA has a direct negative inotropic effect, the indirect hypercapnia-mediated sympatho-adrenal effects of increased heart rate and decreased afterload lead to a net increase in cardiac output [45]. It is somewhat reassuring to note that HCA can increase tissue oxygen delivery in moderate to severe ARDS, as in this study [44].

Finally, a systematic review in 2013 [46] evaluated the effect of low tidal volumes at initiation of mechanical ventilation on the risk of developing ARDS. Twelve observational studies and one randomized controlled trial (RCT) were included in the analysis. In the only RCT included [47], use of larger tidal volumes was more likely to lead to development of ARDS. Additionally, the majority of the observational data demonstrated increased ARDS incidence with larger tidal volumes. However, as pointed out by the authors, there was a great deal of heterogeneity in the studies, precluding a formal meta-analysis. Adding to the uncertainty in this area is the use of ideal body weight and predicted body weight interchangeably, while predicted body weight has been used to guide tidal volume adjustment in most studies. This study and others highlight the fact that the ideal approach to ventilate patients without ARDS is unknown.

Two recent multicenter RCTs were not included in this systematic review. The first study concluded that the use of low tidal volumes for ventilation during surgery improves postoperative outcomes [48]. However, this study used low tidal volume, PEEP and recruitment maneuvers, versus conventional tidal volumes without PEEP, in patients who were deemed high risk for developing pulmonary complications [48]. In contrast, the most recent study - PROVHILO (Protective Ventilation using High versus Low positive end-expiratory pressure) trial [49] - concluded that high PEEP and recruitment maneuvers did not protect against postoperative pulmonary complications but led to an increased incidence of hypotension intraoperatively.

## Non-invasive ventilation and respiratory failure

Non-invasive ventilation (NIV) can reduce intubation and mortality rates in patients with severe acute exacerbation of chronic obstructive pulmonary disease [50] or cardiogenic pulmonary edema [51]. The role of NIV in patients with ARDS is controversial, not least because lung-protective ventilation, a strategy with an 8.8% absolute reduction in the risk of death, is difficult to apply in this setting [2]. Previous studies have shown that NIV applied in patients with ARDS avoids intubation in 54% of the treated patients, with best efficacy in mild ARDS [52]. However, this result could be related to the experience of the center where it is implemented. Antonelli and colleagues [52] also reported that a Simplified Acute Physiology Score II of more than 34 calculated 24 hours after the admission to the ICU and arterial partial pressure of oxygen/fraction of inspired oxygen (PaO_2_/FiO_2_) of not more than 175 after the first hour of NIV are independently associated with the need for endotracheal intubation, and the ICU mortality rate is significantly higher in those who require intubation.

Two recent studies addressed the question of the risks and benefits of NIV in a more mixed population of ICU patients: one was an observational cohort study that looked at intubation rates and outcome in patients presenting to ICU with hypoxemic respiratory failure [53], and the other was an RCT of NIV after weaning of mechanical ventilation and extubation in patients initially presenting with hypoxemic respiratory failure [54].

Thille and colleagues [53] prospectively studied 113 patients receiving NIV for respiratory failure, 82 of whom had ARDS and 31 who had respiratory failure from other causes. Intubation rates were significantly higher in patients with ARDS (61% versus 35%), and NIV failure was highest among those having a PaO_2_/FiO_2_ of less than 150 mm Hg. Importantly, ICU mortality rate did not differ according to the time to intubation. Previous studies have indicated that NIV failure in patients with acute respiratory failure is independently associated with poor outcome as compared with patients intubated without prior NIV [55]. As such, the study by Thille and colleagues is reassuring. In this study, as in that of Antonelli and colleagues [52], there was a low risk of NIV failure in patients with mild ARDS, and almost all patients with severe ARDS required intubation. The grey area appears to be moderate ARDS, where Thille and colleagues [53] and Antonelli and colleagues [52] agree that a PaO_2_/FiO_2_ ratio cutoff of 150 mm Hg (20 kPa) appeared to more accurately segregate patients who failed from those who were successfully treated with NIV.

Ornico and colleagues [54], in an RCT, studied the use of NIV versus oxygen mask immediately after extubation in a mixed population of patients with respiratory failure. The use of NIV post-ICU extubation also remains a contentious area, with earlier ‘prophylactic’ use seemingly preferential to treatment of established respiratory failure in this group of patients. There is, however, no consensus regarding the optimal time period to provide NIV after extubation, and the findings of meta-analyses [56] reflect and reinforce the uncertainty over the use of NIV in this area. The findings of Ornico and colleagues support the suggested benefit of NIV in a mixed respiratory failure population immediately after extubation. The use of NIV in treating established respiratory failure after extubation may not be effective and could be harmful [57].

Finally, a study by Oto and colleagues [58] compared the application of continuous positive airway pressure (CPAP) and ventilation via a nasal mask with that of a full face mask, during unconsciousness induced by general anesthesia. Nasal CPAP was more effective in maintaining upper airway patency in unconscious subjects and produced more effective tidal volume. This study has implications for the practice of emergency mask ventilation in the unconscious subject, which is carried out almost exclusively by full face mask ventilation. Nonetheless, it must be emphasized that the efficacy of any CPAP mask - nasal or full face - depends on head and jaw position during unconsciousness.

## Diagnosis and prognosis in acute respiratory distress syndrome and respiratory failure

Patient heterogeneity - that is, the fact that patients with ARDS have a wide spectrum of disease severity as well as markedly different underlying pathophysiology (for example, sepsis versus trauma) - has been, and continues to be, a hallmark of ARDS and respiratory failure populations within clinical trials. This problem remains a major impediment to defining a responsive patient population for a specific intervention, and this issue remains a major unmet medical need in ARDS clinical trial design. Since the diagnosis of ARDS is based on a combination of clinical, oxygenation, hemodynamic, and radiographic criteria, most studies include a highly heterogeneous group of patients. Even severe hypoxemia, the cardinal feature of ARDS, does not reliably delineate disease severity or predict the development and progression of the syndrome or response to treatment in any given patient. The PaO_2_/FiO_2_ ratio is the hallmark for assessing hypoxemia in patients with ARDS. However, current ARDS definitions do not mandate a standardized procedure for its measurement despite our awareness that changes in PEEP and FiO_2_ alter the PaO_2_/FiO_2_ [59,60]. This concern is highlighted in two recent observational reports in which the PaO_2_/FiO_2_ at ARDS onset was incapable of separating patients into distinct categories of severity associated with significantly different mortalities [61,62]. However, a persistently low PaO_2_/FiO_2_ is associated with a poor outcome and may be a marker of failure to respond to conventional therapy [5,6]. This limitation (that is, our inability to define a more homogeneous group of ARDS patients with similar disease severity) may explain why in the last 14 years since the publication of the ARDSnet trial, only two RCTs have had positive results [3,63]. In both trials, only patients with a PaO_2_/FiO_2_ threshold under a specific level (150 mm Hg, 20 kPa) that persisted 18 to 36 hours were enrolled. Thus, a standardized method for assessing lung injury severity must be mandatory to identify a homogeneous group of patients with ARDS.

### Biomarkers

A number of strategies have been proposed to deal with the heterogeneity problem in ARDS trials. One approach is the use of biomarkers to define more homogenous subsets. Biomarkers linked to the mechanism of action of the treatment would be ideal to either identify the subset or monitor response to therapy. Although various putative biomarkers have been investigated in the context of ARDS, their correlation with disease development and disease outcome has been inconsistent. In this respect, two studies deserve consideration. De Luca and colleagues [64] and de Kretser and colleagues [65] evaluated potentially important biomarkers in infant ARDS and in a mixed population of patients with acute respiratory failure, respectively. De Luca and colleagues [64] evaluated the role of elevated levels and activity of secretory phospholipase A2 (sPLA2) in bronchoalveolar lavage (BAL) fluid in infants with ARDS as well as the consequences of its elevation, including increased free fatty acid levels and reduced quantity and quality of surfactant proteins. sPLA2 activity was correlated with surface tension, compliance and oxygenation, as well as clinical outcomes, including pediatric ICU stay, duration of mechanical ventilation, and oxygen therapy. This study identifies sPLA2 as both a potential target and a marker of disease severity in this population.

The second study by de Kretser and colleagues [65] addresses one of the challenges of biomarker research: the difficulty of validating a diagnostic biomarker of ARDS or any other cause of respiratory failure. The ideal marker is one that can predict the development of disease in ‘at-risk’ patients and also distinguish patients with true non-cardiogenic pulmonary edema from those with congestive heart failure, bilateral pneumonia, lymphangitic carcinomatosis, and all other causes of bilateral lung infiltrates and hypoxemia. Thus, at a minimum, a biomarker identified in medical-surgical ICU patients ‘at risk’ of ARDS needs to be validated in ‘at-risk’ trauma patients and also in patients with non-ARDS causes of respiratory failure. The data of De Kretser and colleagues indicate that the activins A and B, members of the transforming growth factor-beta superfamily, are not useful in distinguishing different subtypes of respiratory failure. However, elevated levels of these proteins are linked to poor outcomes, including risk of death.

### Aids for diagnosis in respiratory failure

Two studies focused on methods for enhancing diagnostic sensitivity in respiratory failure. Using a retrospective design, Yoo and colleagues [66] identified the causes of diffuse pulmonary infiltrates in 214 cancer patients admitted to the ICU for respiratory failure. Invasive diagnostic tests such as BAL and transbronchial and surgical lung biopsy increased diagnostic accuracy and altered clinical care. BAL exclusively provided etiologic diagnoses in one third of patients; lung biopsy simultaneously performed with BAL increased diagnostic yield. This is in contrast to previous studies that demonstrated that the additional diagnostic yield of BAL in combination with non-invasive tests is relatively low [67]. The study by Yoo and colleagues [66] reaffirms the importance of bronchoscopy and biopsy for the diagnosis of diffuse pulmonary infiltrates, at least in the immunocompromised patient subgroup.

The relative ease of bedside ultrasound (US) examination and the availability of user-friendly, inexpensive, portable equipment have made chest ultrasonography an interesting alternative method for diagnosis of respiratory disease. In some studies, chest ultrasonography has been shown to be more sensitive in detecting pneumothorax than chest x-ray performed on a supine patient [68]. The marked increase in use of bedside US in recent years has led to the publication of a number of trials evaluating this technology against chest radiography. Alrajab and colleagues [69] conducted a meta-analysis of the available literature that included high-quality articles, avoiding studies that evaluated populations with known pneumothorax and studies that used verification methods other than chest radiography or CT. The pooled sensitivity of US in this study was lower than in previous analyses (78.6% versus 88%) but remained superior to that of chest radiography or CT, thus confirming the accuracy of US for the diagnosis of pneumothorax, particularly in the setting of trauma.

### Assessment of disease severity in respiratory failure

Although a significant number of patients with respiratory failure die or require prolonged mechanical ventilation, the tools for predicting mortality and morbidity in this group of patients are limited [70]. As a high-cost technology (that is, nursing and medical care), mechanical ventilation is increasingly scrutinized because of the increased focus on improving cost efficiency and documenting patient outcomes. Unfortunately, our current ability to accurately assess practices and patient outcomes from assisted ventilation is hindered by wide variations in standard practices and considerable disagreement among physicians regarding many aspect of ventilatory management. Altered lung mechanics and abnormal gas exchange are hallmarks of impaired lung function in ARDS and are of prognostic significance [71], although assessment of lung mechanics does not form part of the Berlin definition of ARDS. The presence of persistent shock, renal failure, increased age, immunosuppression, underlying cause of lung injury, and overall severity of illness were previously identified as important non-pulmonary outcome determinants [72]. However, poor discrimination by current prediction models in observational cohorts suggests that unmeasured factors may account for failure to wean and increased mortality in ventilated patients [73].

A number of studies in 2013 attempted to improve our prediction of severity of disease, duration of mechanical ventilation, and mortality from respiratory failure. Supinski and Callahan [74] studied the effects and etiology of diaphragm weakness in mechanically ventilated patients on outcome, including mortality and need for long-term ventilation. The authors used an objective measure of diaphragm strength, bilateral anterior magnetic phrenic nerve stimulation using esophageal and gastric pressure sensors, to capture maximal transdiaphragmatic pressure with bilateral stimulation of the phrenic nerves. They found that mortality was 49% in the patients with the weakest diaphragms but was only 7% for patients with mild diaphragm weakness. In addition, patients with the weakest diaphragms took longest to wean from mechanical ventilation, which was a better predictor of failure to wean than other indices of lung dysfunction. Finally, in this study, evidence of infection was a predictor of strikingly lower levels of diaphragm strength than that observed for non-infected patients [74].

Several authors who have studied diaphragm strength invasively have reported that patients with greater diaphragm strength are more likely to successfully wean than patients with weaker diaphragms [75,76]. Animal and limited human studies have found that anti-oxidants and physical activity can block or attenuate ventilator-induced diaphragm dysfunction by short-term mechanical ventilation use [77], but more work investigating cellular and functional changes in the human diaphragm following mechanical ventilation is needed.

A separate study described a simple bedside index, the ventilatory ratio (VR), which uses minute ventilation and PaCO_2_ to calculate an index of ventilatory efficiency [78]. VR was an independent predictor of mortality in a general ICU population and was associated with worse outcome after adjusting for Acute Physiology and Chronic Health Evaluation II score. The advantage of VR is that it is a quick bedside index that identifies patients with severe disease, and the study shows that it is clinically useful in mechanically ventilated patients. Further confirmatory studies are required, however, in a cohort of patients with respiratory failure only.

In another study designed to assess severity of disease during mechanical ventilation, Al-Rawas and colleagues [79] attempted to use the expiratory time constant to provide a real-time measure of inspiratory plateau pressure (Pplat) and respiratory system compliance. Pplat is a surrogate of respiratory system compliance (when PEEP and tidal volume are specified), and monitoring Pplat may be helpful during lung-protective mechanical ventilation. However, it is difficult to measure in patients who are breathing spontaneously or who are on pressure support. Al-Rawas and colleagues [79] found that the expiratory time constant method was an excellent predictor of plateau pressure, compliance, and resistance for acute respiratory failure patients receiving various modes of ventilatory support.

Carlucci and colleagues [80] studied the relationship between patient ventilator asynchrony, a factor known to predict poor outcome in ICU ventilated patients and poor tolerance of NIV, and respiratory mechanics in patients enrolled in a home ventilator program. The occurrence of asynchrony was not correlated to respiratory mechanics recorded during spontaneous breathing and did not differ between patients with obstructive or restrictive disease. As the incidence of asynchrony was high at 30%, other factors, such as ventilator settings, may explain patient ventilator asynchrony in this population.

Kushimoto and colleagues [81] evaluated the relationship between ARDS severity (as determined by the Berlin definition) and extra-vascular lung water (EVLW) and pulmonary vascular permeability, as assessed by the transpulmonary single thermodilution method. ARDS progression outlined by the Berlin definition was associated with increases in EVLW content and pulmonary microvascular permeability. The Berlin definition also distinguished the severity categories of ARDS with good predictive validity for mortality, the severity of physiological derangements, and organ failure [81].

Finally, two studies outlined the factors associated with poor outcome during rescue therapy for ARDS. Camporota and colleagues [82] studied physiological predictors of survival in patients who required high-frequency oscillatory ventilation (HFOV), whereas Aubron and colleagues [83] determined the factors associated with outcome in patients on extra-corporeal membrane oxygenation (ECMO) support. The study by Camporota and colleagues came before publication of the OSCILLATE (Oscillation for Acute Respiratory Distress Syndrome Treated Early) [84] and OSCAR (Oscillation in ARDS) [85] trials, two large multicenter trials of HFOV in ARDS that showed no difference in survival between patients ventilated with HFOV or conventional mechanical ventilation, and in fact, in OSCILLATE, showed an increased risk of death in the HFOV group. The study by Camporota and colleagues is interesting as an early improvement in PaO_2_/FiO_2_ ratio was a predictor of survival at 30 days; patients in this cohort did not survive if there was no improvement in gas exchange within 3 hours [82]. The evidence from the two large trials of HFOV has led to a re-evaluation of the use of this therapy in patients with ARDS. A more individualized approach, using the knowledge gained from studies such as the one by Camporota and colleagues, which identifies responders to HFOV combined with the assessments of cardiac function such as echocardiography, could be incorporated into future HFOV protocols to try to increase the safety of adult HFOV.

The surprising failure of HFOV to show an outcome benefit in ARDS in these studies has led to increased focus on extra-corporeal technologies as rescue therapy for severe ARDS. The study by Aubron and colleagues [83] identified bleeding as the single most frequent and most important complication in patients undergoing ECMO, whereas the volume of blood transfused on veno-arterial ECMO, or the platelet volume requirement on veno-venous ECMO, was an independent risk factor for death. Further studies may indicate whether management of bleeding and coagulopathy can impact on outcome in this severely ill cohort of patients.

## Promising interventions for acute respiratory distress syndrome

Despite 159 RCTs and 29 meta-analyses on ARDS treatment, only three specific interventions have been found to decrease ARDS mortality [1]. The available evidence seems to support a reduction in overall mortality with low tidal volume ventilation and also with prone positioning and neuromuscular blockade among patients with severe ARDS. These three interventions may be the only ones that can be currently recommended for routine clinical use. It must be noted that the survival benefit of these specific interventions has been demonstrated in only a single RCT for each intervention [2,3,63], without any further validation or confirmatory trial. In 2013, investigators evaluated these and other interventions in systematic reviews, pilot studies, and RCTs in an effort to consolidate the supportive evidence and to identify new interventions for this devastating syndrome.

A *post hoc* analysis [86] of the ARDS Network low tidal volume study [2] found that the lower the plateau pressure, the better the patient’s chances of survival. These and other data [34] have prompted the use of even lower tidal volumes in an effort to provide additional benefit in ARDS, with the use of extra-corporeal, pumpless arterio-venous approaches to CO_2_ removal [87]. Forster and colleagues [88] studied the effects of low-flow CO_2_ removal integrated into a renal-replacement circuit in 10 patients who had ARDS and acute kidney injury and who underwent renal replacement therapy. This low-flow hollow-fiber gas exchanger implemented in a renal-replacement circuit led to a rapid, partial, or complete correction of the pH and a significant reduction of the partial pressure of CO_2_ within 4 hours. A marked reduction of vasopressor needs and an improved hemodynamic stability occurred in five of six unstable patients. This small proof-of-concept study must be followed by larger controlled studies in order to assess the impact of low-flow CO_2_ removal on ventilator management and patient prognosis.

In marked contrast to this study, Spieth and colleagues [89] tested the efficacy of ‘noisy’ pressure support ventilation in a randomized crossover study of 13 mechanically ventilated patients with respiratory failure. Noisy pressure support ventilation delivers a random variation of pressure support to the patient in an attempt to mimic normal tidal ventilation. All of the patients in this study were already on assisted spontaneous breathing prior to the start of the study. This mode of ventilation was found to be safe and to result in similar gas exchange and hemodynamics when compared with conventional pressure support and was associated with improved patient-ventilator synchrony compared with conventional pressure support ventilation. Improved synchrony has been well documented during assisted mechanical ventilation modes that apply pressure support proportionally to the inspiratory effort, like Proportional Assist Ventilation and Neurally Adjusted Ventilator Assist [90,91]. The importance of these modes in improving clinically important outcomes remains to be tested.

Finally, two systematic reviews evaluated emerging supportive treatments for ARDS - neuromuscular blockers [92] and ECMO [93] - both treatments that have not yet been incorporated into standard ARDS management worldwide. Alhazzani and colleagues [92] analyzed the combined effects of RCTs that administered a 48-hour infusion of cisatracurium besylate to patients with ARDS on mortality, ICU and hospital stay, duration of mechanical ventilation, and ICU-acquired weakness. Analysis from three pooled trials involving 431 patients demonstrated improvement in mortality without an increase in the risk of ICU-acquired weakness. As these trials were derived from a single group of investigators at multiple centers across France, further international multicenter trials are warranted to confirm the generalizability of these findings.

Zangrillo and colleagues’ [93] meta-analysis of ECMO for H1N1-induced ARDS included observational studies only. They analyzed 266 patients from eight studies and suggested an overall in-hospital mortality of 27.5%, a median ICU stay of 25 days, and an overall median total length of stay of 37 days. However, the highly variable outcomes among the included studies, with in-hospital or short-term mortality ranging between 8% and 65%, suggest the need for additional randomized studies in this area to more accurately define the factors associated with positive and negative outcomes during ECMO for ARDS.

## Conclusions

Progress in specific treatments for ARDS beyond lung-protective strategies of mechanical ventilation and conservative fluid management has not yet been realized. To develop novel therapies, we need to improve our ability to define appropriate molecular targets for preclinical development and, using relevant animal models and human models, develop better methods to determine the clinical value of novel ARDS agents. Clinical trials must have meaningful endpoints and use the available observational and meta-analytic data to inform design. Biomarker-driven studies or defined ARDS subsets should be considered to categorize specific ‘at risk’ populations most likely to benefit from a new treatment. These innovations have been evident in the past year in respiratory critical care research, in laboratory studies, in observational research that attempts to better define diagnosis and prognosis, in interventions aimed at defining further benefit that can be gained from protective lung ventilation strategies, and in the evaluation of new therapies for the treatment of this devastating syndrome.

## Note

This article is part of a collection of Year in review articles in *Critical Care*. Other articles in this series can be found at http://ccforum.com/series/Yearinreview2013

## Abbreviations

ARDS: Acute respiratory distress syndrome
BAL: Bronchoalveolar lavage
CO_2_: Carbon dioxide
CPAP: Continuous positive airway pressure
CT: Computed tomography
ECMO: Extra-corporeal membrane oxygenation
EVLW: Extra-vascular lung water
HCA: Hypercapnic acidosis
HFOV: High-frequency oscillatory ventilation
IL: Interleukin
NIV: Non-invasive ventilation
OSCILLATE: Oscillation for acute respiratory distress syndrome treated early
PaCO_2_: Arterial partial pressure of carbon dioxide
PaO_2_/FiO_2_: Arterial partial pressure of oxygen/fraction of inspired oxygen
PDGF: Platelet-derived growth factor
PEEP: Positive end-expiratory pressure
Pplat: Inspiratory plateau pressure
RCT: Randomized controlled trial
sPLA2: Secretory phospholipase A2
US: Ultrasound
VILI: Ventilator-induced lung injury
VR: Ventilatory ratio
